# Pediatric thoracic outlet syndrome proven by arterial blood pressure monitoring during general anesthesia: a case report

**DOI:** 10.1186/s40981-020-00361-4

**Published:** 2020-07-16

**Authors:** Hidekazu Ito, Shoji Mizuno

**Affiliations:** Department of Anesthesiology, Aichi Developmental Disability Center Central Hospital, 713-8 Kagiya-cho, Kasugai-City, Aichi 480-0392 Japan

To the Editor,

Thoracic outlet syndrome (TOS) is a group of disorders that occur when blood vessels or nerves in the space between the clavicle and the first rib (thoracic outlet) are compressed [1]. While TOS usually occurs in women and young adults, we experienced a pediatric case during general anesthesia.

A 9-year-old boy (height 127 cm, weight 18.6 kg) was bedridden due to sequelae of neonatal-onset medium-chain acyl-CoA dehydrogenase deficiency (MCADD). Femoral osteotomy and selective muscle release of the hip and the knee were planned for hip dislocation and joint contracture. The procedure was successfully completed under general and epidural anesthesia with standard monitoring and invasive arterial blood pressure (ABP) monitoring of the left radial artery. When both upper extremities were abducted to fix the cast on the lower extremities, the ABP suddenly decreased from 77/37 to 29/23 mmHg. Hypovolemia was suspected, and a bolus of hydroxyethyl starch was administered. While a 180 mL (10 mL/kg) bolus was not effective, the ABP improved when the left upper extremity was returned to its place. Since this phenomenon was repeatedly recognized, we performed left axillary artery ultrasonography. The results are shown in Fig. 1. The pressure waveform of the radial artery was lost only when the left upper extremity was abducted with external rotation, resulting in a diagnosis of TOS. No complications occurred during the emergence from general anesthesia, and the patient returned to the general ward.

**Fig. 1:**
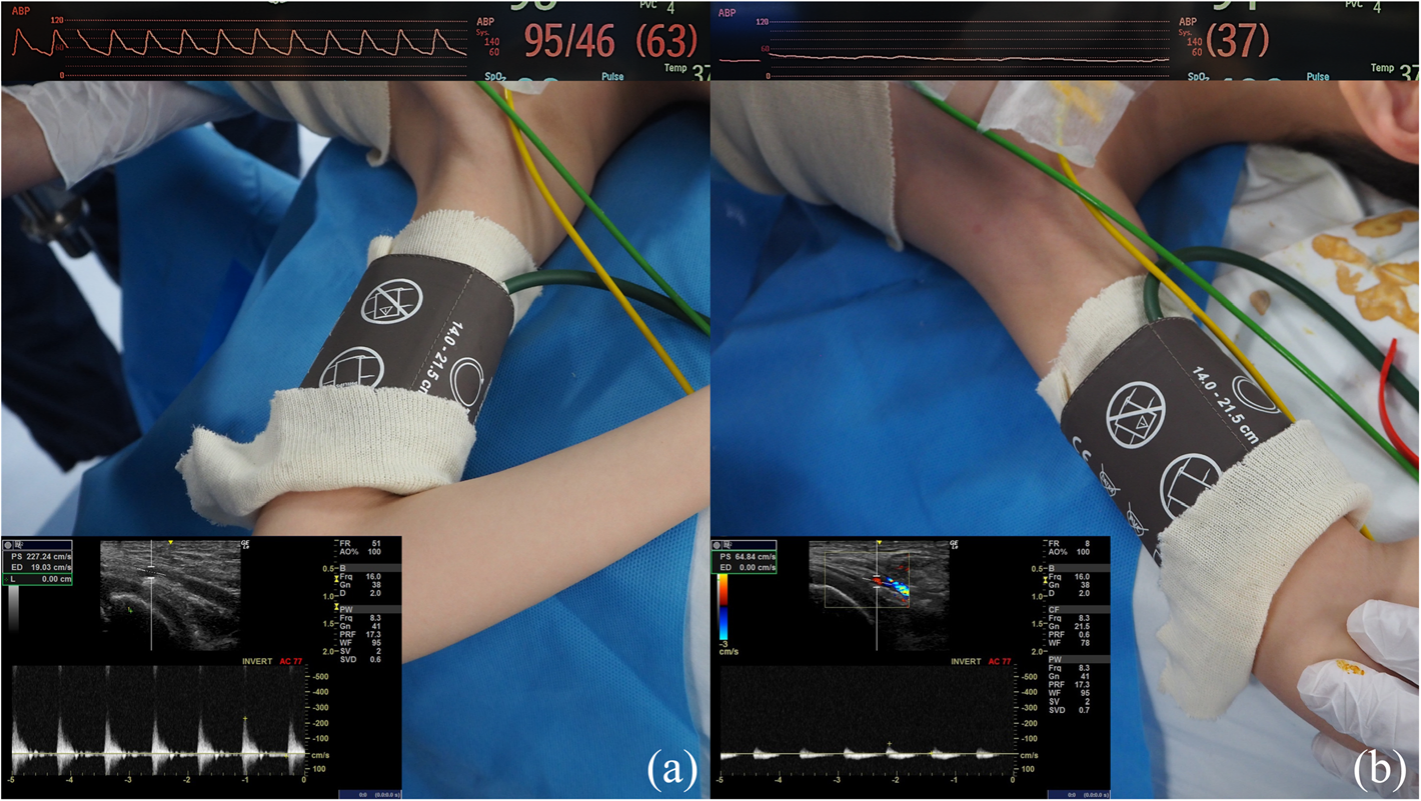
Change in the radial and axillary arterial flow. **a** Slightly abducted position: the pressure waveform of the radial artery and Doppler signal of the axillary artery exist. **b** Excessive abducted position with external rotation: the pressure waveform of the radial artery and Doppler signal of the axillary artery have disappeared

TOS is divided into three types as follows: (i) neurogenic, (ii) vascular, and (iii) non-specific [1]. The signs and symptoms of neurogenic TOS include numbness or pain on the upper extremities and weakening grip. In contrast, symptoms of vascular TOS include discoloration, pain, swelling, and weak or no pulse in the affected upper extremities. The lack of a Doppler signal of the axillary artery indicates vascular TOS. Neurogenic TOS may also exist, as the subclavian artery and brachial plexus run through the same “thoracic outlet”. Since the present patient is lean and lacks supporting tissue due to the sequelae of MCADD, the range of the shoulder joint motion has expanded.

The diagnosis of vascular TOS with ultrasonography has yet to be established. The typical findings include a lack of a Doppler signal and increased echogenicity of the vascular lumen on grayscale images [2]. However, these findings may also be seen in normal patients. In addition, his inability to communicate verbally made it impossible to assess his symptoms. While computed tomography and magnetic resonance imaging were not performed because of no surgical indication, the lack of the pressure waveform of the radial artery and a Doppler signal of the axillary artery strongly suggested vascular TOS.

The upper extremities are sometimes abducted during pediatric general anesthesia. Pediatric anesthesiologists should take special care to avoid excessive abduction with external rotation to prevent postoperative neurological defects. In cases without ABP monitoring, pediatric anesthesiologists need to observe the pulse oximetry waveform of upper extremities carefully and consider the possibility of TOS when it disappears.

## Data Availability

Please contact the first author to obtain the data of this article.

## Abbreviations

TOS: Thoracic outlet syndrome
MCADD: Medium-chain acyl-CoA dehydrogenase deficiency
